# A Simple but Accurate Method for Evaluating Drug-Resistance in Infectious HCVcc System

**DOI:** 10.1155/2017/1236801

**Published:** 2017-08-22

**Authors:** Jian-Rui Li, Wen-Jing Li, Jun-Jun Cheng, Meng-Hao Huang, Zhou-Yi Wu, Chen-Chen Jiang, Hu Li, Jin-Hua Chen, Xiao-Qin Lv, Biao Dong, Jian-Dong Jiang, Zong-Gen Peng

**Affiliations:** ^1^Institute of Medicinal Biotechnology, Chinese Academy of Medical Sciences and Peking Union Medical College, Beijing 100050, China; ^2^State Key Laboratory of Bioactive Substance and Function of Natural Medicines, Institute of Materia Medica, Chinese Academy of Medical Sciences and Peking Union Medical College, Beijing 100050, China

## Abstract

Use of direct-acting antivirals sometimes causes viral drug resistance, resulting in inefficiency in treated patients in real-world practice. Therefore, how to rapidly and accurately evaluate drug resistance is an urgent problem to be solved for rational use and development of antivirals in the future. Here, we aim to develop a new method by which we can evaluate easily but effectively whether a drug will still be efficient in the future treatment in infectious hepatitis C virus cell culture system. HCV-infected Huh7.5 cells were treated with drugs and the culture supernatants were replaced with fresh culture media containing the same drugs at 24 hours. The supernatants were harvested at 48 hours and incubated with naïve Huh7.5 cells. Intracellular HCV RNAs or proteins in the newly infected cells were extracted and analyzed at 48 hours or longer. Results showed that after being treated with telaprevir mutant viruses were easily detected which were resistant to telaprevir, while after being treated with sofosbuvir drug-resistant viruses did not emerge. In conclusion, the new method is simple and quick but accurate to evaluate whether a drug will be still efficient in the forthcoming therapeutic regimen and whether drug resistance will occur after long-term treatment with drugs.

## 1. Introduction

Hepatitis C virus (HCV) infection causes chronic hepatitis, liver fibrosis, cirrhosis, and even liver cancer, which is considered a major serious threat to people's health worldwide [1, 2]. In the past few years, several direct-acting antiviral agents (DAAs) were approved for use in clinic, including NS3/4A protease inhibitors, NS5B polymerase inhibitors, and NS5A protein inhibitors [3–6], and the overall therapeutic effect was largely improved. However, monotherapy with DAAs often induces drug-resistant HCV variants, resulting in a rebound or failure in treated patients [7–10]. To overcome it, recently, five fixed-dose combined agents were approved for clinic use, including Harvoni, Viekira Pak, Technivie, Zepatier and Epclusa, which contain NS5A inhibitor plus NS5B and/or NS3/4A inhibitor(s). Treatment with those combined agents indeed improved the overall sustained viral response (SVR) to >90% in HCV-infected patients [11–13]. However, the overall therapeutic effect is still suboptimal in real-world practice, and the consequence are partly caused by the emergence of drug-resistance [14, 15]. So, how to quickly evaluate whether an infection will be resistant to novel antiviral agents is a very important consideration to correctly use and develop antiviral agents in the coming future.

Up to now, there are many HCV mutants reported which are resistant to known approved DAAs [16]. Once the mutants were detected, the DAAs will be inefficient for the patients. However, some new mutations will emerge which will also be resistant to known DAAs or new compounds. Currently, HCV sub- or full-length genomic replicon or infected cells were commonly used to analyze drug-resistance profile of DAA [17], and there are several methods to evaluate the emerged drug-resistance [17]. The first method is selection of mutant viruses. After the cells were treated with drugs for a long experimental time, intracellular HCV RNA was extracted, and the sequences encoded HCV enzymes were amplified and cloned into an expression vector. Single-colonies were selected and the nucleotide sequences of HCV RNA were sequenced, then the enzyme proteins (HCV NS3 or NS5B) were prepared, and the drugs were evaluated in the cell-free system with it for the drug-resistance profile. Or the treated replicon cells were diluted and single cell-colonies were directly selected out and subcultured, then HCV RNAs were extracted, and nucleotides were sequenced to evaluate the drug-resistance [18, 19]. The second method is induced-resistance in replicon or infected cells. The dose of antiviral agent was gradually increased and thus resistant viruses were induced, but it takes a relatively long time as well [20, 21]. The third is a direct-sequencing in which the nucleotides of HCV RNA were directly sequenced by a next-generation sequencing technology [22, 23]; though it is rapid, the problem of false positive is inevitable, and thus it also needs a relatively long time to be further verified for the new variants. Here, we developed a new method for rapid and accurate evaluation of drug-resistance in infectious HCV cell culture (HCVcc) system, which can be used to analyze whether a drug will be still effective in the forthcoming therapeutic regimen.

## 2. Materials and Methods

### 2.1. Cells and Virus

Huh7.5 cells and the plasmid pFL-J6/JFH/JC1 containing a full-length chimeric HCV complementary DNA (cDNA) were kindly provided by Vertex Pharmaceuticals Inc. (Boston, MA). Huh7.5 cells were cultured in Dulbecco's Modified Eagle Medium (DMEM, Invitrogen, CA) supplemented with 10% inactivated fetal bovine serum (Invitrogen) and 1% penicillin-streptomycin (Invitrogen). HCV virus stock was prepared as previously described [24]. Briefly, after the full-length HCV RNA was prepared, it was transfected into Huh7.5 cells with Lipofectamine 2000 (Invitrogen), and the culture medium was collected as HCV viral stock. Huh7.5 (HCV+) cells were HCV-positive Huh7.5 cells which were infected with HCV for over 10 days.

### 2.2. Agents

Compounds sofosbuvir (PSI-7977), telaprevir (VX-950), and simeprevir were purchased from MedChemExpress (Princeton, NJ). The monoantibody (mAb) to HCV Core (ab2740) or NS3 (ab13830) was from Abcam, Co. Ltd. The mAb to beta-actin (TA-09) was from Beijing ZSJQ-BIO Co. Ltd. Goat anti-mouse (sc-2005) and goat anti-rabbit (sc-2004) secondary antibodies were from Santa Cruz Biotechnology Inc.

### 2.3. Selecting Mutant Viruses

Huh7.5 (HCV+) cells were seeded into 25-cm^2^ cell culture flask. After having been incubated for 24 hours, the cells were treated with 1 *μ*M of simeprevir, 10 *μ*M of sofosbuvir, or solvent control, respectively, and concentration was over 10 times the half maximal effective concentration (EC_50_) against wild type (WT) HCV in Huh7.5 (HCV+) cells. After having been treated 24 hours, the cells were washed and continuously incubated with fresh culture media that contained simeprevir or sofosbuvir at the same concentration. The culture supernatants, which contain drugs and progeny viruses secreted from Huh7.5 (HCV+) cells, were harvested in 48 hours and then directly incubated naïve Huh7.5 cells. Intracellular HCV RNAs in the newly infected cells were extracted with a RNeasy Mini Kit (Qiagen) at 48 hours after infection, and total intracellular proteins were extracted using a CytoBuster Protein Extraction Reagent (Novagen) with 1 mM protease inhibitor cocktail (Roche) after the newly infected cells were passaged 1 to 3 times.

### 2.4. Cloning and Sequencing

The RNAs extracted from newly infected cells were reverse-transcribed into cDNA using a GoScript Reverse Transcription System (Promega). The sequence of HCV NS3/4A or NS5B was amplified using a PfuUltra II Fusion HS DNA Polymerase (Agilent) with the primers (5′-CGGAATTCATGGCTCCCATCACTG-3′ and 5′-GCTCTAGATCAATGGTGATGGTGATGATGGCATTCCTCCATCTCAT-3′ (for HCV NS3/4A) or 5′-CGCGGATCCATGTCCATGTCATACTCCTGGACCG-3′ and 5′-GGCGAATTCCGGTTAGCGCGACAC-3′ (for HCV NS5B)). The PCR production was then constructed into a vector pcDNA3.1(+). After the plasmids were transformed into competent cells Trans 5*α*, more than 60 clones were randomly selected out and sequenced.

### 2.5. Constructing Infectious HCV with Site-Directed Mutagenesis and Verifying Its Drug-Resistance

Mutant plasmids, such as pHCV-D168V and pHCV-S282T, were constructed by Taihe Biotechnology Company (Beijing, China) with a site-directed mutagenesis method. The mutant sequences were identified with sequencing. An infectious virus stock was prepared as WT type. Huh7.5 cells were seeded into 96-well or 6-well plates at a density of 3 × 10^4^ cells/cm^2^. After having been incubated 24 hours, the cells were treated with drugs and were simultaneously infected with HCV virus stock (wild or mutant type). After 72 hours, intracellular RNAs or proteins were extracted and were then quantified with a real-time one-step quantitative reverse-transcription polymerase chain reaction (qRT-PCR) or western blot (WB), respectively.

### 2.6. qRT-PCR

The total RNA extracted from cells was analyzed using an AgPath-ID One-Step RT-PCR Kit (Applied Biosystems, Foster, CA). Fluorescent signals were detected with a 7500 fast real-time PCR system (Applied Biosystems) according to the manufacturer's procedure with the primers and probes (primers 5′-CGGGAGAGCCATAGTGGTCTGCG-3′ and 5′-CTCGCAAGCACCCTATCAGGCAGTA-3′ and probe FAM-5′-AGGCCTTGTGGTACTGCCT-3′-TAMRA for detecting HCV, primers 5′-CGGAGTCAACGGATTTGGTCGTAT-3′ and 5′-AGCCTTCTCCATGGTGGTGAAGAC-3′ and probe FAM-5′-CCGTCAAGGCTGAGAACGG-3′-TAMRA for internal control gene glyceraldehyde 3-phosphate dehydrogenase (GAPDH)). All quantifications were normalized to the level of GAPDH, the levels of HCV RNA were analyzed with a 2^−ΔΔCT^ method, and a half-maximal effective concentration (EC_50_) was calculated with the Reed-Muench method as previously [25].

### 2.7. Western Blot

Western blot (WB) was performed as previously described [25]. Briefly, after the membrane was washed, the target proteins were accordingly probed with the antibody to HCV Core (1 : 2500) or NS3 (1 : 2500). As an internal control, the antibody to actin (1 : 4000) was used. After having been washed with TBST, the membrane was incubated with the goat anti-mouse or goat anti-rabbit secondary antibody, respectively. The proteins were detected using an Immobilon Western Chemiluminescent HRP Substrate (Millipore, Inc.) with ChemiDo XRS gel imager system (Bio-Rad, CA). The protein signal intensity was scanned with the Gelpro32 software, and a ratio of the interested protein to the internal control protein actin was calculated and normalized as 1.00 for the control group.

### 2.8. Statistical Analysis

Data shown in histograms or in tables were means ± standard deviation from over 3 independent experiments and were analyzed using an* ANOVA* analysis and* Student's t-test*. The level of significance was set at *P* < 0.05.

## 3. Results

### 3.1. Mutant Viruses Were Selected out after Treatment with a High Concentration of Drug in HCV Infectious System

For selecting mutant viruses to evaluate the drug-resistant profile, HCV-positive (HCV+) Huh7.5 cells were treated with high concentration of simeprevir or sofosbuvir for 24 hours and then washed and continuously incubated with fresh culture media containing the same drugs for additional 48 hours. The culture supernatants were harvested and directly incubated naïve Huh7.5 cells. Then intracellular HCV RNAs were extracted in 48 hours and reverse-transcribed into cDNA, and then the full-length NS3/4A or NS5B sequence was amplified and cloned into a vector. Sequencing results showed that at least one amino acid mutant was identified in each HCV NS3/4A clone from being treated with 1 *μ*M of simeprevir when compared with the amino acid sequences from being treated with solvent control (WT type) (Table 1). A mutation with D168V was detected with the highest frequency (98.3%), followed with R343Q (78.3%) and Y56H (46.7%). A mutation with V102A, K122N, R147K, S281C, G304A, T312R, I354T, A390T, S398P, Y475C, G498E, M581T, K583E, or V666A was detected with <5% mutation frequency (Table 1). However, multiple-site mutations were also observed and were almost associated with the D168V mutation (Table 1).

However, the NS5B sequence was not amplified after treatment with 10 *μ*M of sofosbuvir, suggesting that no drug-resistance virus was emerged, so we decreased the drug's concentration and used 1 *μ*M of sofosbuvir which is equal to the EC_50_ against WT HCV in Huh7.5 (HCV+) cells. After the sequences were cloned and were analyzed, at least one amino acid substitution in the HCV NS5B sequence was identified. A mutation H118R was detected with the highest frequency (96.7%), followed with E333G (71.7%) and N335S (71.7%). A mutation D55G, V435A, or T273A was observed with <5% mutation frequency (Table 2). Multiple-mutations were also detected (Table 2), and, interestingly, mutations of E333G and N335S were always combined, and almost all of the multiple-site mutations were related to the mutation H118R (Table 2).

### 3.2. Drug-Resistances Were Validated in the Newly HCV-Infected Huh7.5 Cells

**Figure 1:**
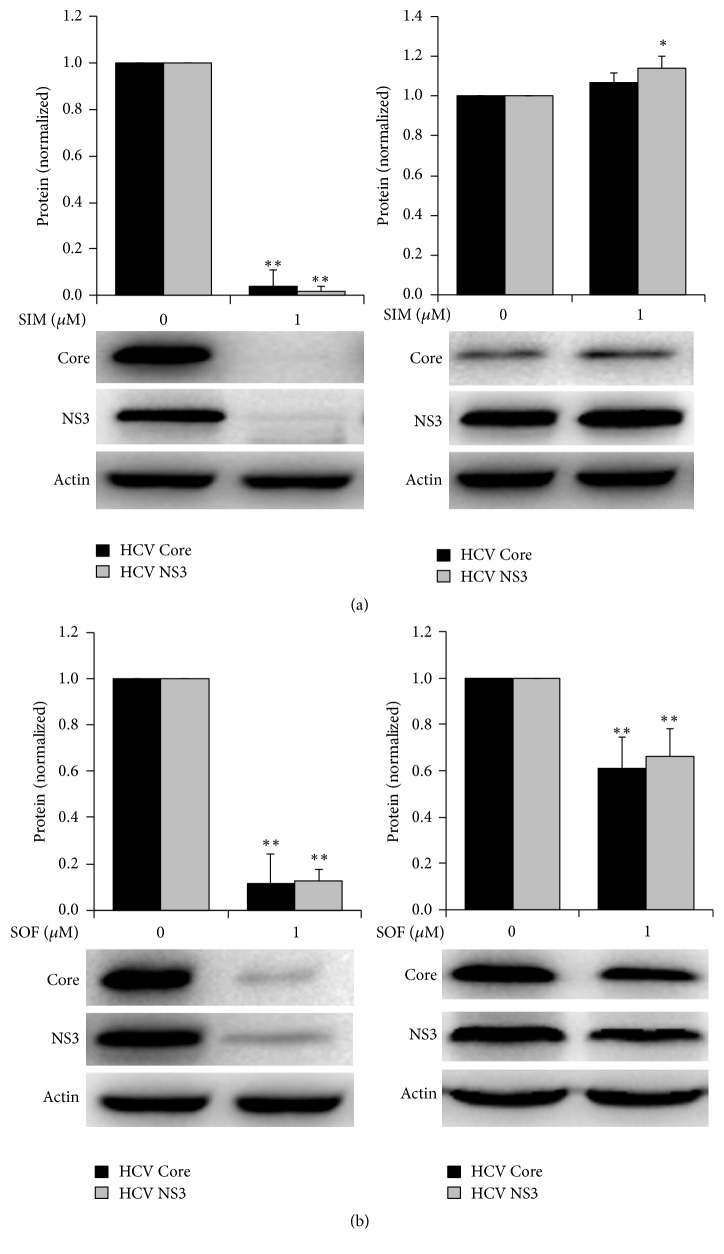
*Verification of drug-resistant viruses*. Huh7.5 (HCV+) cells were treated with 1 *μ*M of simeprevir ((a), right) or sofosbuvir ((b), right) or solvent control ((a) and (b), left). At 24 hours, the cells were washed and continuously incubated with fresh culture media containing drugs again for 48 hours. The cultural supernatants were then harvested and directly incubated to naïve Huh7.5 cells. After having been passaged 1~3 times, the newly infected cells were treated with 1 *μ*M of simeprevir (a) or sofosbuvir (b) for 72 hours. Intracellular proteins were extracted and detected with WB. ^*∗*^*P* < 0.05 and ^*∗∗*^*P* < 0.01 versus solvent control. All of the experiments were performed for over 3 times. The bands presented in the figure were from a representative experiment. Presented is mean ± standard deviation and Student's* t*-test was used.

To analyze whether the progeny viruses secreted from drug-treated Huh7.5 (HCV+) cells will be resistant to the original drugs, the newly HCV-infected Huh7.5 cells, which were incubated with the infectious supernatants harvested from drug-treated Huh7.5 (HCV+) cells, were passaged 1~3 times with fresh culture media and then treated with 1 *μ*M of simeprevir or sofosbuvir again. After 72 hours, intracellular proteins were extracted and were detected. Results showed that the progeny viruses (WT HCV) secreted from solvent-control-treated Huh7.5 (HCV+) cells were very sensitive to simeprevir (Figure 1(a), left) or sofosbuvir (Figure 1(b), left). But the inhibitory activities against progeny HCV secreted from simeprevir-treated cells were completely disappeared when treated with simeprevir again (Figure 1(a), right), while those secreted from sofosbuvir-treated cells remained sensitive when treated with sofosbuvir again (Figure 1(b), right). Results suggest that the progeny viruses in supernatants from simeprevir-treated cells were resistant to simeprevir, while those from sofosbuvir-treated cells were still sensitive to sofosbuvir.

### 3.3. Drug-Resistant Mutants Were Confirmed in the Naïve Huh7.5 Cells

**Figure 2:**
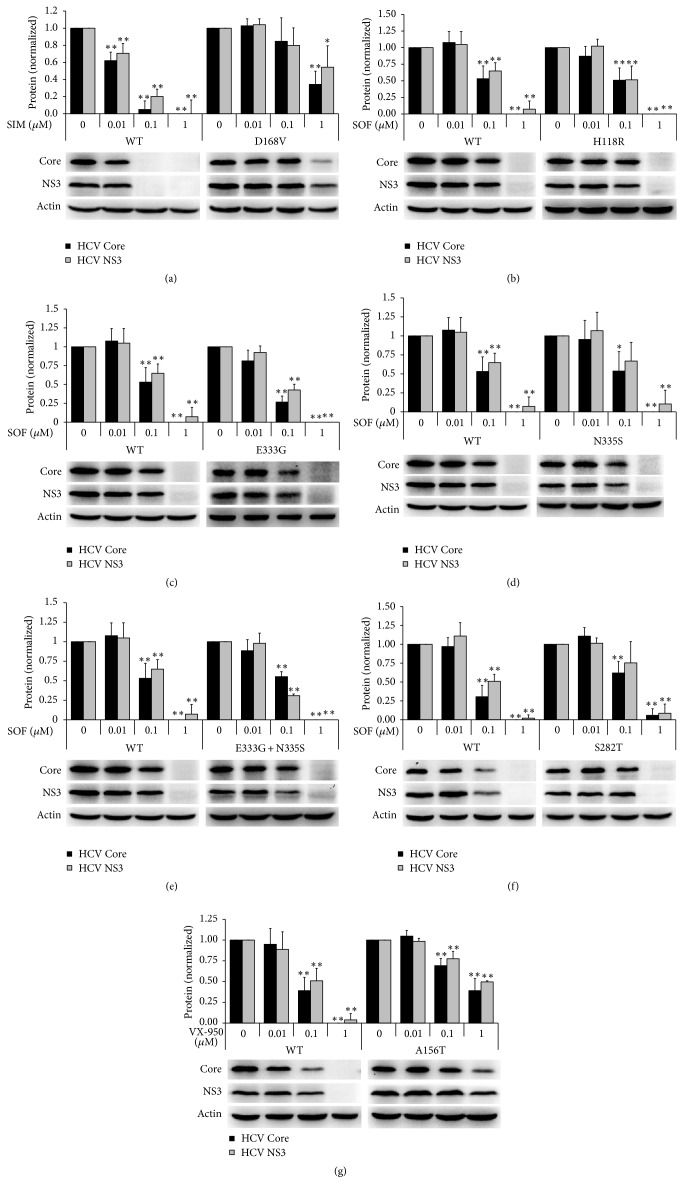
*The anti-HCV activity of drugs against WT and mutant HCV in Huh7.5 cells*. Naïve Huh7.5 cells were infected with wild and mutant type HCV and simultaneously treated with drugs. Intracellular proteins were extracted and detected with WB in 72 hours. (a) Simeprevir against WT and D168V mutant HCV. ((b)–(f)) Sofosbuvir against WT or H118R (b), E333G (c), N335S (d), E333G + N335S (e), or S282T (f) mutant HCV. (g) Telaprevir (VX-950) against WT and A156T mutant HCV. ^*∗*^*P* < 0.05 and ^*∗∗*^*P* < 0.01 versus solvent control. All of the experiments were performed for over 3 times. The bands presented in the figure were from a representative experiment. Presented is mean ± standard deviation and Student's* t*-test was used.

To further analyze whether the mutation in viruses will lead to drug-resistance, we selected several mutations with relatively high mutation frequency and constructed them into the full-length virus plasmid with a site-directed mutagenesis method. After infectious virus stocks were prepared and their infectivity was detected, naïve Huh7.5 cells were infected with HCV virus stock and simultaneously treated with antiviral agents; after 72 hours, intracellular HCV RNAs and proteins were extracted and detected with qRT-PCR and WB, respectively. As shown in Table 3, the EC_50_ of simeprevir against the D168V mutant virus was 0.94 *μ*M, increasing 94-fold when compared with that against the WT HCV (Table 3). The resistance profile was verified at protein levels (Figure 2(a)). However, the EC_50_ of sofosbuvir against H118R, E333G, N335S, or E333G/N335S mutant virus was not increased when compared with that against the WT HCV (Table 3, Figures 2(b), 2(c), 2(d), and 2(e)). This agrees with our results detected in the newly infected Huh7.5 cells (Figure 1(b)), and the result suggested that sofosbuvir is a drug with high genetic barrier to drug-resistance [26].

Though the S282T or A156T mutation was reported to have resistance to sofosbuvir or telaprevir, respectively [27, 28], the mutation was not observed in our experiment after being treated with sofosbuvir or simeprevir. However, we still constructed the mutation viruses and evaluated their resistance to telaprevir, simeprevir, or sofosbuvir. Result showed that the EC_50_ of sofosbuvir against the S282T mutant virus was increased 6.5-fold when compared with that against the WT HCV (Table 2), and the result was confirmed at protein levels (Figure 2(f)). Certainly, the S282T variant was sensitive to simeprevir (Table 3). The EC_50_ of telaprevir (VX-950) against the A156T mutant virus was increased 54-fold when compared with that against the WT HCV (Table 2), and the drug-resistance profile was also confirmed at protein levels (Figure 2(g)). However, the A156T mutant virus was still sensitive to simeprevir and sofosbuvir (Table 3), and in our system the A156T mutant was also not observed after treatment of simeprevir.

## 4. Discussion

In this study, we developed a new method for quick and accurate evaluation of drug-resistance in viral infectious system. When HCV-infected Huh7.5 cells were treated with a high concentration of DAAs for about 3 days, only the progeny viruses that already harbor mutations conferring resistance to the drug can survive and replicate in the newly incubated Huh7.5 cells, which can be indicated by detecting HCV RNA or/and proteins conveniently. Compared with traditional or present methods [18–23], this method has several advantages for evaluating drug-resistance. Firstly, it is simple and thus saves the time and resources for quick evaluating whether a drug will be still effective and whether an infection will be resistant to DAAs, especially for evaluating drug-resistance to unknown mutant HCVs. Secondly, it is accurate. A drug will possibly be inefficient if HCV RNAs or proteins can be detected in the assay. On the contrary, if the HCV cannot be detected, the drug will be with high genetic barrier to the virus, because only the drug-resistant viruses can propagate in the next newly infected Huh7.5 cells, and the propagation of HCV will be further limited by the high concentration of drugs in the supernatants harvested from drug-treated Huh7.5 cells (HCV+). Thirdly, the method can be used to evaluate all of antiviral agents that targeted not only replicative enzymes but also entry and excretion steps, while using replicon cells is restricted to drugs that only targeted intracellular replicative steps.

In our experiments, the D168V mutant was selected out after treatment of simeprevir, which is consistent with the literature reports about the drug-resistance profile of simeprevir [29, 30], while the A156T mutant was not observed, and the resistance was validated by the treatment of simeprevir and telaprevir (Table 3), suggesting that the A156T mutant virus was still sensitive to simeprevir and sofosbuvir, which agrees with the clinical report [31, 32]. Usually, double or multiple mutant strains should be observed under drug pressure compared to wild type strain with no drug. In our result, multiple-site mutations were also observed and were almost associated with the D168V mutation (Table 1) after treatment of simeprevir, suggesting that D168V mutation is the main occurrence that induces drug-resistance. Also, after treatment with low concentration of sofosbuvir, mutations were always combined, and almost all of the multiple-site mutations were related to the mutation H118R (Table 2). The significance of it is worth further investigation. However, the S282T mutation with low drug-resistance was not observed after being treated with sofosbuvir, which was reported previously in cell cultures of HCV genotype 2b [10, 33]. It might be a cause of different virus strain, which is HCV genotype 2a in our system, while previous report was used with non-genotype 2a [28]. Certainly, we also verified that the mutation S282T is low resistant to sofosbuvir in our experiments, consisting with the recent report [10]. Furthermore, despite treatment with a low concentration of sofosbuvir, variants were selected out; however, they remained sensitive to sofosbuvir, and the results also validated that sofosbuvir is a drug with high genetic barrier to virus [34]. All those data suggested our method can accurately evaluate the profile of drug-resistance. However, more experiments are needed to validate the method with other HCV genotypes and more DAAs, especially with viruses from clinical patients.

Because HCV replication in hepatocytes is very fast and the HCV NS5B polymerase is lacking a correction mechanism during HCV RNA replications, there exit simultaneously many mutant viruses in patients. Several studies have demonstrated that mutant HCV variants result in resistance to NS3/4A, NS5A, or NS5B inhibitors, and the mutant viruses preexist in HCV-infected patients who have not been previously treated with DAAs [35–38]. So guidelines from the most important medical associations have been currently recommending the analysis of Q80K mutation in NS3/4A in patients, which has high prevalence of this naturally occurring variant for genotype 1a and its clear association with decreased SVR after simeprevir based therapy [39]. Thus, it is of great significance to quickly evaluate whether DAAs will be helpful to a patient and reduce the risk of invalid or rebound use after treatment in the coming future. Recently, Saeed et al. constructed a cell line that is persistently expressed SEC14L2, and it allows pangenotype and clinical HCV to be naturally infected [40]. Our current data suggested that it may be suitable if the special HCVcc system is available; however, this needs to be further verified. If combined with this method, it will be more convenient to evaluate whether use of the given drugs will be rebound or invalid in patients in the coming therapeutic regimen. Furthermore, this method may be theoretically widely used to rapidly and accurately evaluate the drug-resistance in other viral infectious systems besides HCV; however, more experiments are needed to further verify its catholicity.

## 5. Conclusion

In this study, we developed a new method, by which we can easily analyze whether a drug will be still effective in the forthcoming therapeutic regimen. It is simple and quick but accurate when compared with traditional or recent methods. It may be also widely used to evaluate the drug-resistance in other viral infectious systems.

## Figures and Tables

**Table 1:** Mutations in NS3/4A sequence after treatment with simeprevir.

Mutation	Frequency
D168V	59/60
R343Q	47/60
Y56H	28/60
A390T	3/60
G304A	2/60
V102A	1/60
K122N	1/60
R147K	1/60
S281C	1/60
Q309R	1/60
T312R	1/60
I354T	1/60
S398P	1/60
Y475C	1/60
G498E	1/60
M581T	1/60
K583E	1/60
V666A	1/60
Y56H/D168V	4/60
Y56H/T312R	1/60
Y56H/D168V/S281C	1/60
Y56H/D168V/R343Q	1/60
D168V/R343Q	7/60
D168V/A390T	1/60
D168V/V102A	1/60
D168V/G304A	1/60
D168V/Q309R/R343Q	1/60
D168V/I354T/S398P	1/60
D168V/R343Q/Y475C	1/60
D168V/R343Q/G498E	1/60
D168V/M581T/K583E	1/60
D168V/R343Q/K122N/V666A	1/60

**Table 2:** Mutations in NS5B sequence after treatment with sofosbuvir.

Mutation	Frequency
H118R	58/60
E333G	43/60
N335S	43/60
T273A	3/60
D55G	1/60
S79N	1/60
V435A	1/60
R337T	1/60
E333G/N335S	43/60
H118R/T273A	2/60
H118R/V435A	1/60
H118R/D55G	1/60
H118R/S79N	1/60
H118R/T273A/E333G/N335S/R337T	1/60

**Table 3:** Anti-HCV activities of drugs against WT and mutant HCV in Huh7.5 cells.

HCV	Inhibitor	EC_50_ (*μ*M)	Changed fold
WT	Telaprevir	0.01 ± 0.01	—
WT	Simeprevir	0.01 ± 0.01	—
WT	Sofosbuvir	0.08 ± 0.04	—
D168V	Simeprevir	0.94 ± 0.85	94.0
H118R	Sofosbuvir	0.03 ± 0.05	0.38
N335S	Sofosbuvir	0.01 ± 0.01	0.13
E333G	Sofosbuvir	0.01 ± 0.01	0.13
E333G/N335S	Sofosbuvir	0.02 ± 0.01	0.25
A156T	Telaprevir	0.54 ± 0.37	54.0
A156T	Simeprevir	0.01 ± 0.01	1.0
A156T	Sofosbuvir	0.11 ± 0.16	1.4
S282T	Sofosbuvir	0.52 ± 0.37	6.5
S282T	Simeprevir	0.01 ± 0.01	1.0

The anti-HCV activities of drugs against wild type (WT) and mutant HCV were detected in Huh7.5 cells infected with HCV 2a. The EC_50_ was calculated with the Reed-Muench method after intracellular HCV RNA was detected with qRT-PCR. Data presented are means ± standard deviation from over 3 independent experiments. Changed fold was the ratio of the EC_50_ of WT against that of mutant HCV.
